# Left Ventricular Flow Analysis: Recent Advances in Numerical Methods and Applications in Cardiac Ultrasound

**DOI:** 10.1155/2013/395081

**Published:** 2013-04-17

**Authors:** Iman Borazjani, John Westerdale, Eileen M. McMahon, Prathish K. Rajaraman, Jeffrey J. Heys, Marek Belohlavek

**Affiliations:** ^1^Department of Mechanical and Aerospace Engineering, University at Buffalo, State University of New York, Buffalo, NY 14260, USA; ^2^Department of Internal Medicine, Division of Cardiovascular Diseases, Mayo Clinic, Scottsdale, AZ 85259, USA; ^3^Department of Chemical and Biological Engineering, Montana State University, Bozeman, MT 59717, USA

## Abstract

The left ventricle (LV) pumps oxygenated blood from the lungs to the rest of the body through systemic circulation. The efficiency of such a pumping function is dependent on blood flow within the LV chamber. It is therefore crucial to accurately characterize LV hemodynamics. Improved understanding of LV hemodynamics is expected to provide important clinical diagnostic and prognostic information. We review the recent advances in numerical and experimental methods for characterizing LV flows and focus on analysis of intraventricular flow fields by echocardiographic particle image velocimetry (echo-PIV), due to its potential for broad and practical utility. Future research directions to advance patient-specific LV simulations include development of methods capable of resolving heart valves, higher temporal resolution, automated generation of three-dimensional (3D) geometry, and incorporating actual flow measurements into the numerical solution of the 3D cardiovascular fluid dynamics.

## 1. Introduction

The key function of the heart is to maintain blood circulation. This is accomplished through repetitive cycles of systolic contraction and diastolic relaxation. Systole and diastole consist, however, of several component phases that impact intraventricular flow. In a normally functioning heart, following isovolumic contraction (during which myocardial tension increases, but both aortic and mitral valves are closed), the aortic valve opens, and the LV ejects blood into the aorta. As a result of the helical architecture of its fibers [1], the LV twists during the systolic cycle, stores part of its kinetic energy as potential energy that is to be released later as elastic recoil (untwisting), and supports early diastolic suction [2]. The diastolic cycle starts with an isovolumic relaxation phase, during which the aortic valve is already closed, but the mitral valve is not yet open. Untwisting of the LV generates a pressure gradient that allows for suction of blood into the chamber and leads to mitral valve opening [2]. A filling jet of blood flow rushes into the LV, generating a diastolic vortex [3]. As the pressure gradient across the mitral valve equilibrates, the transmitral flow slows down, but this period of diastasis can hardly be considered as a complete stagnation of the blood  [4, 5]. Left atrial contraction drives the remainder of LV filling during the latter part of diastole. An intraventricular flow vortex changes its strength and location during the course of diastole; however, its duration has been observed beyond the time point of mitral valve closure [3]. Studies suggest that kinetic energy stored in the flow vortex contributes to both timely closure of the mitral valve and blood flow redirection towards the outflow tract [6, 7].

Thus, the LV is not a simple positive displacement pump [8], and although the flow inside the LV is quite complex and multidirectional, the cycles of systole and diastole essentially generate a flow continuum controlled by interactions between the LV wall and blood mass. The shape, size, and dynamics of the LV and other cardiac chambers, proper mechanical function of the cardiac valves, ventriculoaortic coupling [9, 10], negative or positive inotropic drugs [2], and neurohumoral effects are some of the various factors that affect heart function and, thus, intraventricular blood flow. Consequently, understanding fluid dynamics inside the LV and other cardiac chambers is critical to identify subtle cardiovascular disorders in their early stages, optimize treatment of a dysfunctional or failing heart, develop better ventricular assisting devices or artificial hearts, and further advance the designs of prosthetic heart valves—to name only a few examples.

**Figure 1:**
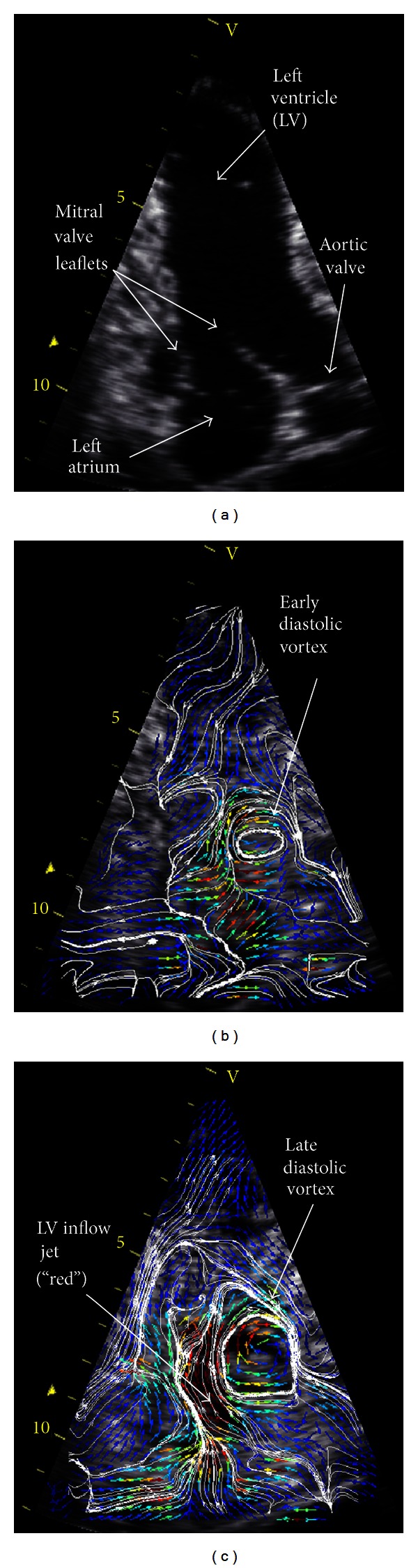
Echo particle image velocimetry study of normal heart. (a) Epicardial echo scan. Notice the well-defined LV boundary. Anatomic structures are labeled. After injection of microbubbles, digital transfer, and offline tracking, (b) early and (c) late diastolic inflows and vortex are mapped by velocity vectors and isovelocity streamlines. Blue, green, and red colors indicate low, mid, and high flow velocities, respectively.

Ventricular flow can be studied using clinical imaging techniques and image-based computational fluid dynamics (CFD). Ventricular flow measurements using clinical imaging have been summarized elsewhere [11]. In this review, we focus on CFD approaches, the image-based data required for CFD from the experiments, and finally combining flow obtained from the CFD methods with cardiac ultrasound flow measurements (Figure 1). This review paper is organized as follows. In Section 2.1, we present numerical methods for flow simulations based on the motion of the LV boundary. In Section 2.2, we focus on ultrasound imaging and review current options for analysis of boundary conditions and blood flow tracking inside cardiac chambers, with particular attention to the emerging echo-PIV. In Section 2.3, the methods for combining the flow field obtained from CFD and experimental measurements are discussed. In Section 3.1, we critically review the previous work on simulations of the LV, and we discuss limitations and summarize future research in Section 3.2. 

## 2. Materials and Methods

### 2.1. Numerical Methods for Simulating LV Flow Based on LV Wall Motion

Ventricular flow is mainly driven by motion of the myocardium, a dynamically evolving surface boundary inside the LV. A major challenge in the numerical simulation is to satisfy the boundary conditions on the LV surface that is continuously changing in time. The methods for handling large deformations/movements of the boundary can be broadly categorized into two classes: (a) boundary-conforming techniques; and (b) fixed grid techniques. In boundary-conforming techniques the grid moves with the moving boundary, whereas, in fixed grid techniques, the grid is not moving and effects of the moving boundaries are transferred onto the closest fixed grid nodes that are in the vicinity of the moving boundary. The boundary-conforming techniques can retain good grid resolution near the moving boundaries. Consequently, they typically require fewer grid points relative to the fixed grid techniques to achieve the same level of resolution near the moving boundaries. Large boundary movements, however, in the boundary-conforming approaches create highly skewed grids that reduce the convergence and accuracy of the numerical scheme [12, 13]. To avoid highly skewed grids, computationally expensive remeshing of the grid may be required [12–15]. In what follows we examine these two classes of methods in more detail. 

#### 2.1.1. Boundary-Conforming Techniques

In boundary-conforming methods the computational grid is fitted to and moves/deforms with, the moving boundary. The grid movement is taken into account in the formulation of the Navier-Stokes equation by incorporating the grid velocity terms, which is referred to as the Arbitrary Lagrangian-Eulerian (ALE) formulation [16]. A critical condition for accurate solutions of the ALE equations is to satisfy the geometric conservation law in discrete form [17–19]. The method, which was implemented in the commercial software STAR-CD, has been applied to simulate blood flow in the LV using geometrical information obtained from magnetic resonance imaging (MRI) images [20]. However, due to the low frame rate of the MRI images (10 frames per cycle) the LV geometry reconstruction was approximate and did not provide a smooth flow curve. Furthermore, Cheng et al. [21] used the ADINA commercial software with the ALE formulation to simulate the filling phase of an LV with a simplified geometry. This simulation, due to the simplified geometry and wall properties, did not yield a realistic LV motion [21].

The ALE method works well for problems with relatively simple geometries and moderate deformations, such as those encountered in compliant blood vessels. However, obtaining smooth computational meshes at every time step for problems with significant structural deformations is difficult, and frequent remeshing may be the only option [14, 15]. For instance, Saber et al. [20] created 1100 meshes from an original set of ten MRI images using linear interpolation. Such mesh creation is quite time consuming and computationally expensive. Due to these inherent difficulties, the ALE method is not the most attractive option for simulating ventricular flows, and most studies have employed fixed grid techniques. 

#### 2.1.2. Fixed Grid Techniques

The fixed grid techniques are of particular interest in problems involving large deformations and movements of the boundary. Many fixed grid techniques have been developed in the past decades, including the immersed boundary (IB) and immersed interface methods [22–24], the level set method [25–27], the fictitious domain method [28, 29], and the cut-cell method [30]. In such methods the entire fluid computational domain is discretized with a single, fixed, nonboundary-conforming grid while the immersed solid domain is typically discretized with a separate set of the Lagrangian nodes, which are tracked individually. The effect of a moving immersed body on the fluid is accounted for by adding, either explicitly or implicitly, body forces to the governing equations of motion on the nodes in the vicinity of the immersed body [23]. Identifying the fluid nodes in the vicinity of the solid bodies and calculating body forces, either implicitly or explicitly, add to the computational cost of the method relative to the ALE method. Furthermore, the fixed grid methods need relatively high resolution in the regions where the boundaries are moving. If the movement of the boundaries is large, the size of the high resolution region must also be large, which adds to the computational cost. Nevertheless, these computational costs are typically smaller than the cost of remeshing or moving the mesh with the boundary-conforming methods.

From the different fixed grid methods developed, the immersed boundary, pioneered by Peskin [31–33], was first developed to simulate the flow through the heart and the heart valves. However, the original IB method does not capture the interface as a sharp edge. It smears the interface by distributing the body forces over several grid nodes in the vicinity of the immersed boundary. The fictitious domain method [28] is another diffused interface method, which uses a fixed grid and has been applied to heart valve simulations [34–40]. In this method, which is similar to Peskin's IB method, the immersed solid is free to move within the fluid mesh, but the two domains are coupled together at the solid/fluid interface through a Lagrange multiplier (or local body force) [40]. A major issue with diffuse interface methods is that they cannot yield accurate results for the viscous shear stresses on the solid boundary due to the smearing of the boundary, and excessive resolution is required to achieve accurate shear stresses. To solve this issue, a series of sharp-interface IB methods have been proposed [41–45]. IB methods and their diffuse or sharp-interface variations are reviewed in [23]. The sharp-interface IB methods satisfy the boundary conditions exactly at the position of the immersed boundary and do not have the smearing effect [41–43]. Therefore, the majority of recent LV [7, 46–50] and heart valve [45, 51–56] simulations have been carried out using this method.

### 2.2. Ultrasound Methods for Cardiac Tissue Motion and Flow Measurements

Patient-specific data on cardiac tissue motion and flow measurements is essential for image-based CFD simulations to provide realistic boundary conditions. This is due to the fact that the accuracy and realism of such CFD simulations strongly depend on the specifications along the heart wall boundary, including the location, geometry, and velocity of the heart wall. Such boundary conditions can only be provided by the experimental measurements or a mathematical model of the LV wall. However, mathematical models of the heart wall are still not sufficiently accurate to be used for specific patients [57], and so those approaches are not reviewed. The cardiac flow measurement methods described here are useful for validating CFD simulations and gaining confidence in the validity of the simulated flow field. 

#### 2.2.1. Cardiac Border Tracking

Determination of boundary conditions over the duration of the cardiac cycle requires tracking of the inner (endocardial) border of the muscle defining the analyzed cardiac cavity, such as the LV. Manual delineation of the endocardial border is currently involved in most studies, at least as an initial, user-determined estimate of the boundary at a given time point. Software tools, such as Omega Flow (Siemens) [58] to name only one of the various professional or custom software applications, are available to complete border tracking throughout the rest of the cardiac cycle.

#### 2.2.2. Echo-PIV

*In vivo* visualization of complex spatial features of intracardiac flow can be achieved using echo-PIV, a term initially coined by Kim et al. [59, 60]. Echo-PIV typically utilizes commercially available contrast particles, such as albumin-shell (Optison) or lipid-shell (Definity) microbubbles. Based on the authors' experience, microbubbles of air can serve as echo-contrast particles in some experimental settings as well. The particles are tracked by computer software between individual ultrasound image frames for sequences of high-frame-rate, brightness- (B-) mode, and 2D ultrasonographic images [3, 6]. To our knowledge, echo-PIV utilizing 3D spatial tracking of microbubbles has not been validated at the time of preparing this paper. Computer tracking of microbubble displacement can be accomplished with processing software designed for optical-PIV [6]; however, a software program specific to echo-PIV is currently under development [58]. 

Echo-PIV is minimally invasive (injection or infusion of diluted microbubbles), relatively inexpensive, does not involve ionizing radiation, and offers high temporal resolution combined with suitable 2D spatial resolution, when compared to existing techniques. Information regarding several commonly used intracardiac flow visualization techniques is detailed by Sengupta et al. [11]; however, aside from echo-PIV, only phase encoded MRI is commonly used in CFD analyses for boundary and flow visualization data. Cardiac MRI provides good 3D spatial resolution with the ability to measure a fully 3D velocity field. However, the data are time-averaged over many cardiac cycles, and, therefore, real-time flow patterns, particularly turbulent motion and multidirectional flow, are diminished or lost. Additional limitations include the high cost of both MRI equipment and tests and the long data acquisition time (~20 minutes) [11, 60]. 

The primary constraint of current echo-PIV is the inability to measure out-of-plane particle motion; thus, only 2D velocity fields can be generated. However, recent advancements in 3D ultrasound equipment or the use of multiplanar acquisition techniques may represent a step towards resolving this problem [61]. Characteristics of the ultrasound system available for an echo-PIV analysis will determine both the temporal and spatial resolutions. Recent experimental studies have demonstrated that frame rates as high as 500 fps with an axial resolution of 1.2 mm and a lateral resolution of 1.7 mm are achievable and would allow velocity measurements up to 50 cm/s [62]. Additional limitations include distortion due to the scan conversion process for B-mode images, sector size width (typically less than 45 degrees) and the possible underestimation of high velocities in physiological flows [11, 63]. 

Extensive validation of echo-PIV has been conducted both *in vitro* [59, 60, 62, 64, 65] and *in vivo* [3, 66]. Results have demonstrated that echo-PIV measurements are in close agreement with theoretical and experimental flow analyses, including flow patterns that simulate intraventricular flow [3, 63]. Furthermore, the usefulness of echo-PIV as a diagnostic technique has been demonstrated in a clinical setting. Recently, Faludi et al. [58] demonstrated the ability of echo-PIV to distinguish intraventricular flow patterns in healthy and dysfunctional hearts, noting markedly different flow patterns for patients with implanted mechanical heart valves. They determined that LV energy dissipation was significantly increased in patients with the mechanical valves and theorized that echo-PIV may be used to optimize valve replacement surgery. Additional advancements in clinical applications of echo-PIV include the analysis of the diastolic vortex as an indicator of cardiac health. During the diastolic (i.e., LV filling) phase of the cardiac cycle, formation of a 3D donut-shaped flow vortex has been linked to natural optimization of the intraventricular swirling flow [7]. An example of the intraventricular vortex, visualized *in vivo* using echo-PIV, is shown in Figure 1. 

Another approach for intracardiac flow vortex analysis, which does not require the use of PIV, has been introduced by Gharib and his colleagues [67], who established optimal fluid dynamic conditions for vortex development through quantitative analysis of vortex formation time (VFT) and demonstrated a universal timescale of VFT [68]. They also documented the role of VFT in optimization of biological fluid transport [69] and showed the possible utility of VFT as an index of cardiac health [67]. Using VFT, Kheradvar et al. [70] studied the relationship between transmitral vortex formation and abnormal diastolic filling patterns in patients with diastolic dysfunction and pointed out the importance of better understanding vortex formation. Reports by others have documented that cardiac dysfunction, such as moderate elevation of LV afterload [71, 72] or restrictions of the LV by pericardial adhesions [73], negatively impacts vortex formation conditions. One study with VFT also suggested that patients with Alzheimer's disease may have impaired diastolic filling efficiency compared to age-matched control subjects [74].

### 2.3. Combining Experimental Flow Measurements with Numerical Solutions

As reviewed above, a number of numerical approaches have been developed and applied to the modeling of blood flow in the LV. An obvious question then is why should another method be considered? The weighted least-squares finite element method (WLSFEM) has three relatively unique attributes that make the approach particularly appealing for simulating the complex flows inside the moving LV, especially in settings where patient-specific blood flow data are available. First, with most existing methods, as the computational mesh is refined, the computational time increases roughly quadratically with the number of grid points. The WLSFEM, however, has the potential to offer optimal scalability, that is, the computational time is directly proportional to the number of grid points for any number of points [75–83]. This optimal scalability is due to the fact that the least-squares approach was designed from the beginning to work with multilevel or multi-grid linear solvers such as algebraic multi-grid. As a result, while other methods may require the same (or even less) computational time than the WLSFEM on today's moderately refined grids, the WLSFEM should require less computational time on highly refined meshes due to better scalability.

Second, in the WLSFEM, the approximation problem is written as an optimization problem: the goal is to minimize the value of a functional for a finite element approximation space. The value of the functional is a sharp measure of the error everywhere in the domain [78, 84–90]. The value of this functional can be used to (1) determine regions in which the approximate solution does not satisfy the governing equations of conservation of mass and momentum; (2) generate new computational grids that have more refinement in regions that tend to have larger errors; and (3) compare two approximate solutions and determine the one that better satisfies the governing equations in the functional norm.

The third advantage of the WLSFEM approach is that it enables a straightforward and flexible platform for assimilating experimental data into the process of solving the governing equations [91–93]. As a result, one can obtain an approximate solution that satisfies the model equations and also approximately matches patient-specific data. Another way of stating this advantage is that the WLSFEM approach allows one to use the governing equations (e.g., the Navier-Stokes equations or other non-Newtonian model equations) to interpolate and extrapolate the experimental data into a full 3D field that fills the entire LV. This full 3D data can then be used to calculate accurate flow properties that are not possible to fully calculate with just 2D or 1-dimensional data.

The WLSFEM requires defining new variables and rewriting the Navier-Stokes equations as a system of first-order equations. A number of different first-order systems have been derived by others [78, 85, 89, 90, 94–96], but the focus here is on a system that provides better mass conservation [82]. The improved mass conservation system is based on the identity

(1) $$\begin{array}{c} v\cdot \nabla v=-\omega \times v+\frac{1}{2}\nabla \left(v\cdot v\right), \end{array}$$

where **v** is the dimensionless velocity and **ω** is the negative vorticity (∇×**v**), and the definition of variable *r* is

(2) $$\begin{array}{c} r=\nabla p+\frac{\sqrt{\lambda }}{2}\nabla {\left|v\right|}^{2}=\nabla \left(\frac{\sqrt{\lambda }}{2}{\left|v\right|}^{2}+p\right). \end{array}$$

The following first-order system is now used to replace the Navier-Stokes equations:

(3) $$\begin{array}{c} \nabla \times v+\omega =0\;\;\text{in}\;\;\Omega ,\\ \nabla \cdot v=0\;\;\text{in}\;\;\Omega ,\\ -\lambda \left(\frac{\partial v}{\partial t}+v\times \omega \right)-r+\nabla \times \omega \;=\;0\;\;\text{in}\;\;\Omega ,\\ \nabla \cdot \omega =0\;\;\text{in}\;\;\Omega ,\\ \nabla \times r=0\;\;\text{in}\;\;\Omega ,\\ \nabla \cdot r-\lambda \left(\omega \cdot \omega +v\cdot r\right)=0\;\;\text{in}\;\;\Omega ,\\ v=g_{1}\;\;\mathrm{on}\;\;\partial \Omega_{1},\\ n\times v=g_{2}\;\;\mathrm{on}\;\;\partial \Omega_{2}. \end{array}$$

The WLSFEM approach uses a functional that includes boundary weights:

(4) GPIV(v,ω,r)=||∇×v+ω||Ω2+||∇·v||Ω2+||∇×ω−r−λ(v×ω + ∂v∂t)||Ω2+||∇·ω||Ω2+||∇×r||Ω2+||∇·r−λ(ω·ω + v·r)||Ω2+w1h||v−g1||∂Ω12+w2h||n×v−g2||∂Ω22,

where (*w*/*h*)||·||_0,Γ_^2^ is the weighted *L* ^2^-norm along the 2D boundary surfaces (∂*Ω*_1_) or 2D surfaces where experimental data is given (∂*Ω*_2_). The experimental plane is simply a 2D cross-section that is typically somewhere near the middle of the 3D domain, whereas the other boundaries are all on the surface of the 3D domain. The functions *g*_1_ and *g*_2_ are the given boundary or PIV data that is to be weakly matched by the numerical approximation of the solution. For example, *g*_1_ is set to the surface displacement rate along no-slip boundaries. The spatial location of the PIV data, *g*_2_, does not need to be the same as the computational mesh node locations. The data can be located anywhere within the computational domain.

The boundary functional weights, *w*, should be chosen so that the weight value is larger in the regions where the given data is known more accurately and smaller in the regions where the data contains more noise. In [92] it was shown that the boundary functional weight should be chosen by

(5) $$\begin{array}{c} w\approx \frac{1}{\sigma^{2}}, \end{array}$$

where *σ* is the standard deviation in the given data, which can often be provided by the PIV analysis software or estimated from time series data. 

When modeling blood flow in the LV or any fluid-structure interaction problem, the shape of the fluid domain is continuously changing. Many numerical strategies exist for addressing the changing domain shape, including the generation of a new mesh every time step or grid mapping using equations such as the Winslow generator [83, 97]. Another straightforward method is to solve a compressible elasticity problem over the fluid domain and use the solution from the elasticity problem to move the nodes of the finite element mesh. This approach is often referred to as a pseudosolid domain mapping technique [98, 99].

**Figure 2:**
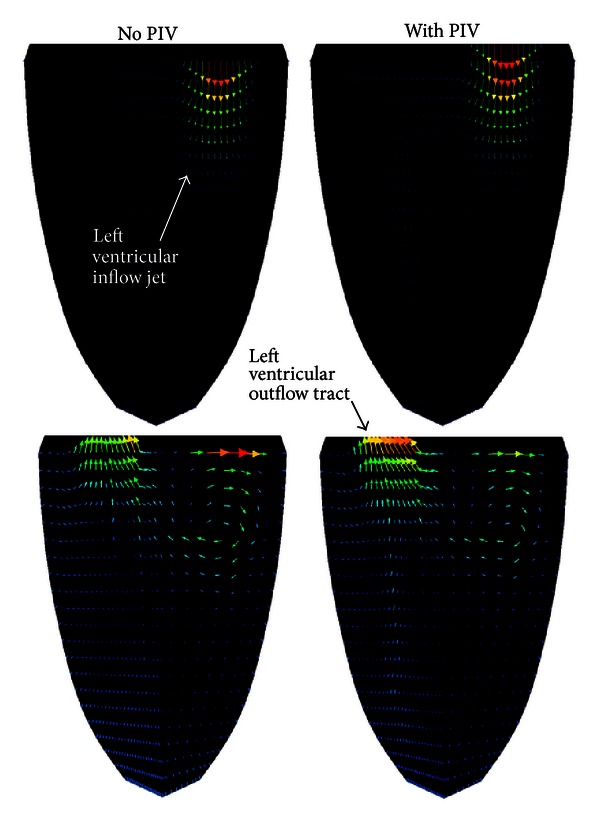
Simulation of a filling flow jet entering the left ventricle (top panels) and flow vortex in the inflow region along with an ejection jet in the left ventricular outflow tract (bottom panels) by using the weighted least-squares finite element method (WLSFEM), without the assimilation of particle image velocimetry (PIV) data (left panels) and with assimilated data (right panels). Blue, green, and red colors indicate low, mid, and high flow velocities, respectively.

The use of the WLSFEM for the simulation of flow in the LV is illustrated in Figure 2 The LV is approximated using a fully 3D ellipsoid geometry with moving wall and details of the mathematical model are available in [91]. Velocity data from echocardiographic PIV can be assimilated into the simulation, and Figure 2 shows the 3D velocity field for a single plane early in the filling stage and later during the ejection stage both with and without assimilated PIV data. Cursory visual inspection of the velocity field reveals only small differences between the simulation with and without the PIV data. This is a good indication that the model is at least somewhat accurate. If the PIV data had a dramatic impact on the velocity field, it would indicate that the model prediction was significantly different from the experimental data. In this case, however, the model prediction is consistent with the experimental data. A detailed inspection of the effect of assimilating the PIV data reveals some small differences. Specifically, the model prediction with the PIV data included has a stronger vortex forming near the middle of the domain during the filling stage and a slightly weaker vortex during the ejection stage.

The development of any mathematical model requires that assumptions are made. In the case of the LV model shown in Figure 2, an assumption was made regarding the shape of the LV, and valves were not included in the model. It is likely that the weaker vortex during filling in the simulation without PIV data is a result of modeling without the valves. As the blood flows through the valve into the LV, drag from the valve flaps would enhance the formation of a vortex. When the PIV data are assimilated into the simulation, it is possible to partially recover the effects of the valve, and the simulation predicts a stronger vortex. In other words, the assimilation of PIV data allows the simulation to partially overcome inaccuracies that are a result of modeling assumptions. This fact makes data assimilation a very attractive technique, whenever experimental data are available.

## 3. Ventricular Fluid Dynamics Simulations and Related Flow Physics

### 3.1. State-of-the-Art

The earliest simulation of ventricular flow dates back to the late 1970s with the pioneering work of Peskin using the immersed boundary method [31]. The heart model was extended to 3D by Peskin and McQueen [33] and to the physiological Reynolds numbers [100]. In Peskin's heart model, the cardiac tissue is modeled as a system of elastic fibers, which add forces to the fluid equations of motion if stretched or contracted. As stated previously, the forces are spread over several grid nodes near the boundary (diffuse interface), which hinders accurate calculations of the shear stress. Furthermore, the motion of the heart in this model is not prescribed based on experimental measurements, and only gross features of the motion are reproduced; that is, patient-specific simulations are not possible with this method. 

Saber et al. [20] carried out the first patient-specific LV flow simulations based on MRI images using the commercial software Star-CD with the ALE formulation. Long et al. [101] also carried out intraventricular flow simulations based on MRI images using the commercial software CFX4 with an ALE formulation and studied the influence of inflow boundary conditions on the solution [101]. They generated meshes with about 54,000 grid nodes for 46 time instants in a cycle from 16 original MRI data [101]. Cheng et al. [21] used the commercial software ADINA with ALE formulation to simulate the flow in the LV. In Cheng et al. [21] the LV has a simplified geometry with about 100,000 elements. All the above simulations, however, could not provide additional insights into the physics of the ventricular flow due to their low temporal and spatial resolutions. The purpose of these early simulations was mostly to show the feasibility of image-based numerical simulations of the LV and not investigating the ventricular flow physics. 

Domenichini et al. [46] carried out 3D simulations of the filling phase in a model with symmetric LV geometry using a sharp-interface immersed boundary method. They found that the dominant flow feature during the filling phase is a vortex structure of a single ring connected to the incomplete wake-induced ring in the boundary layer [46]. In a separate study, these investigators also showed that the physiological placement of the mitral valve creates a vortex during the filling phase that enhances ejection during LV contraction and that an unnatural asymmetry of the inflow could reduce the pumping efficiency of the LV by 10% [7]. They further verified the vortex structure using an experimental model of the LV and comparing their numerical results against the flow measurements [47]. Finally, they showed that the replacement of the natural valve (symmetric inflow toward the center of the LV) with a prosthetic implant (asymmetric inflow) can cause the reversal of the vortical motion in the LV, which can cause higher dissipation of the energy for pumping [49]. Note that in these studies [7, 46, 47, 49] the LV geometry was assumed to be symmetric (prolate sphere) with the prescribed kinematics needed to create a physiological flow waveform. 

Recently, Schenkel et al. [102] used the commercial software Star-CD with ALE formulation to simulate the flow in the LV. In Schenkel el al. [102], the LV geometry for 850 time steps was reconstructed from MRI images (17 frames per cycle) using spline interpolation. Their simulations also show the vortex ring development during the filling phase. However, the calculated flow field was found to be quite sensitive to the inflow boundary conditions [102]. Recent simulations of Zhenga et al. [50] have used the sharp-interface immersed boundary to simulate a simplified, symmetric LV. They have simulated dysfunctional LV cases by changing the ejection fraction ratio in their model and found that such changes affect the vortex dynamics inside the LV. Le and Sotiropoulos [48] have used a sharp-interface immersed boundary method to study the flow in an LV reconstructed from MRI data. They prescribe the motion of the LV based on a cell-based activation methodology, which yields physiologic kinematics [48]. Their simulated flow field shows the development of the mitral vortex ring and the trailing vortex tubes, which originate from the heart wall, and their impingement on the wall at the end of the diastolic phase.

### 3.2. Current Limitations of Simulations

**Figure 3:**
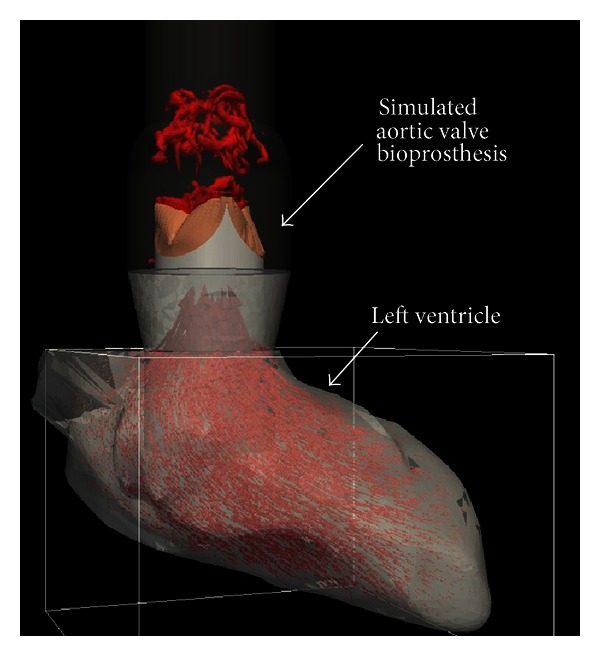
Simulation of the left ventricle and a bioprosthetic heart valve in the aortic position. The 3D vortical structures downstream of the valve are visualized using isosurfaces of q-criteria, whereas the flow inside the LV is visualized using velocity vectors.

As can be observed from the previous section, in all of the LV simulations published to date, the heart valves are not resolved; that is, they are replaced with simplified inflow/outflow conditions. However, several studies have argued the importance and sensitivity of the solution to the inflow condition [101, 102]. Furthermore, the direction (angle) of the inflow has been shown to affect the vortex dynamics inside the heart and even reverse its direction of motion [49]. The heart valves not only affect the direction of the mitral jet into the LV but also can create vortex shedding that affects the vortex dynamics inside the LV. Development of methods capable of resolving the effects of heart valves on the vortex dynamics inside the LV is underway. Specifically, the sharp-interface immersed boundary method [45] over a single curvilinear grid has been extended to overset grids [103] (Figure 3). In overset grids, multiple grids can be arbitrarily overlaid on each other, and the information at their grid interfaces are prescribed by suitable interpolation from the solution of the other grids; for example, in Figure 3 the aorta is discretized with a curvilinear body-fitted grid, while the LV domain is discretized with a Cartesian grid. The LV and heart valves are placed as immersed boundaries onto the overset grids [103]. The motion of the LV is prescribed while the motion of the trileaflet valve is calculated through a fluid-structure interaction coupling [104]. The 3D vortical structures are visualized in the aorta downstream of the valve using the isosurfaces of q-criteria (based on definition, q-criteria identify the 3D vortices where vorticity is higher than strain rate [105]) and using velocity vectors in the LV in Figure 3.

In the simulation pictured in Figure 3, the motion of the LV was prescribed based on a model [48] and not based on experimental measurements. Many other simulations have used simple models instead of patient-specific data as well [7, 32, 33, 46–49]. However, the mathematical modeling of heart motion involves the complex interaction of the electrical excitation, muscle activity, LV tissue properties, surrounding tissue properties, and the blood inside the heart [57]. The mathematical modeling of the heart motion has yet to be performed to a degree of sophistication to handle patient-specific cases, and until that day, they suffer from low accuracy relative to direct measurements of the heart. On the other hand, image-based simulations have typically used MRI data to construct the shape and motion of the LV [20, 101, 102]. However, the temporal resolution of MRI data in these simulations was relatively low (9–17 frames/s) [20, 101, 102]. Echocardiography could be a method of choice to overcome this issue. This imaging technique has considerably higher temporal resolution compared to MRI. Based on the authors' practical experience, depending on the depth and width of scanning, the current clinical echocardiography systems can routinely achieve 100–200 frames/s in a sector B-mode setting.

Another issue that requires attention is automating the generation of a 3D geometry from experimental data. Usually a few cross-sections are available from imaging data, which requires interpolation for the 3D geometry reconstruction from the 2D slices. Currently, most of this process is done manually or semiautomatically in computer-aided design software, which is quite time consuming. Interpolation strategies are required to reconstruct the geometry in time instants, when experimental image frames are not available. Automating this process would save time and minimize or eliminate subjectivity of the currently required user interaction. Finally, the uncertainty in the reconstruction of the 3D geometry from imaging data including cycle-to-cycle LV motion variability, subject motion during the imaging process, and uncertainty in the location of the imaging planes, needs to be quantified to support clinical decision making in the future. 

The simulations to date have used boundary conditions at the LV wall. However, in many cases, simultaneous flow measurements in a few 2D planes are also available. Incorporating the sparse flow measurements into the 3D simulations to augment boundary data will increase the accuracy and realism of the simulations, as suggested in Figure 2 earlier, or enable to incorporate physical and physiological phenomena that are not explicitly included in the mathematical model, such as wall roughness (trabeculation) or valves.

In summary, we propose the following developments to advance the image-based LV simulations.

1.  Methods capable of simulating the LV with heart valves resolved.
2.  Higher temporal resolution for experimental and clinical imaging data.
3.  Automated generation of 3D geometry for CFD analysis from imaging data and quantifying the uncertainty.
4.  Incorporating actual (even if sparse) flow measurements into the numerical solution of the 3D cardiovascular fluid dynamics.
